# Identification of hub genes and transcription factors in patients with primary gout complicated with atherosclerosis

**DOI:** 10.1038/s41598-024-54581-0

**Published:** 2024-02-18

**Authors:** Lu Xiao, Shudian Lin, Feng Zhan

**Affiliations:** 1 Department of Rheumatology and immunology, Affiliated Wuxi Fifth Hospital of Jiangnan University, The Fifth People’s Hospital of Wuxi, Wuxi, Jiangsu China; 2 Department of Rheumatology and immunology, Hainan general hospital (Hainan Affiliated Hospital of Hainan Medical University), Haikou, Hainan China

**Keywords:** Gout, Atherosclerosis, Bioinformatics, Immune infiltration, Transcription factor, Computational biology and bioinformatics, Rheumatology

## Abstract

Evidence shows that primary gout is prone to develop to atherosclerosis, but the mechanism of its occurrence is still not fully clarified. The aim of this study was to explore the molecular mechanism of the occurrence of this complication in gout. The gene expression profiles of primary gout and atherosclerosis were downloaded from the gene expression omnibus database. Overlapping differentially expressed genes (DEGs) between gout and atherosclerosis were identified. The biological roles of common DEGs were explored through enrichment analyses. Hub genes were identified using protein–protein interaction networks. The immune infiltrations of 28 types of immune cells in gout and control samples from GSE160170 were evaluated by the ssGSEA method. Transcription factors (TFs) were predicted using Transcriptional Regulatory Relationships Unraveled by Sentence Based Text Mining (TRRUST) database. A total of 168 overlapping DEGs were identified. Functional enrichment analyses indicated that DEGs were mostly enriched in chemokine signaling pathway, regulation of actin cytoskeleton, and TNF signaling pathway. CytoScape demonstrated 11 hub genes and two gene cluster modules. The immune infiltration analysis showed that the expression of DEGs in gout was significantly upregulated in activated CD4 T cells, gamma delta T cells, T follicular helper cell, CD56dim natural killer cells, and eosinophil. TRRUST predicted one TF, RUNX family transcription factor 1. Our study explored the pathogenesis of gout with atherosclerosis and discovered the immune infiltration of gout. These results may guide future experimental research and clinical transformation.

## Introduction

Gout, one of the most common inflammatory arthritis with high uric acid level as a key characteristic, always results in incapacitating joint pain and poor quality of life^1,2^.The management of gout mainly lies in non-steroidal anti-inflammatory drug and uric acid lowering therapy. The prevalence of gout is increasing year by year, the estimated prevalence was 3 million cases in United State^3^.

The link between no matter hyperuricemia or gout with risk factors for cardiovascular disease is well-documented and consistent. Numerous studies have demonstrated that the gouty patients were more likely to experience coronary heart disease than those without gout^4,5^. Atherosclerosis, a chronic cardiovascular disease, is suggested to be strongly associated with the elevated uric acid level. Atherosclerosis would be strongly prompted by hyperuricemia via regulating inflammatory signaling pathways, including macrophage M1/M2 polarization, CRP, and NLRP3-inflammasomes, in the meantime, the formation of atherosclerotic plaques can be reversed by uric acid lowering treatment^6^. In addition, endothelial dysfunction also plays an important role in gout induced atherosclerosis^7^. Previous study discovered that patients with gout have shorter telomeres than healthy participants. In patients with gout, the number of flares and cardiovascular disease was related to the telomere shortening^8^. This may be the underlying reason for the high incidence of atherosclerosis among gouty patients.

Although a link between atherosclerosis and gout has long been noticed, research as to the specific mechanism of this relationship was neglected. With the help of microarray techniques, the capability to detect the differentially expressed genes (DEGs) among different groups of people have increased. Therefore, in this study, the potential pathogenesis of gout and atherosclerosis was explored via bioinformatics analyses. In addition, the immune infiltration of gout was also detected to further understand the high prevalence of atherosclerosis in gouty patients. The microarray datasets, GSE160170 for gout and GSE28829 for atherosclerosis, were downloaded from the GEO database. Functional enrichment analyses were clustered by DEGs. Furthermore, STRING database was used to constructed a protein–protein interaction (PPI) network. Subsequently, transcription factors (TFs) related to the pathogenesis of gout and atherosclerosis were predicted. Finally, immune infiltrations of gout were investigated.

## Materials and methods

### Data collection

**Figure 1:**
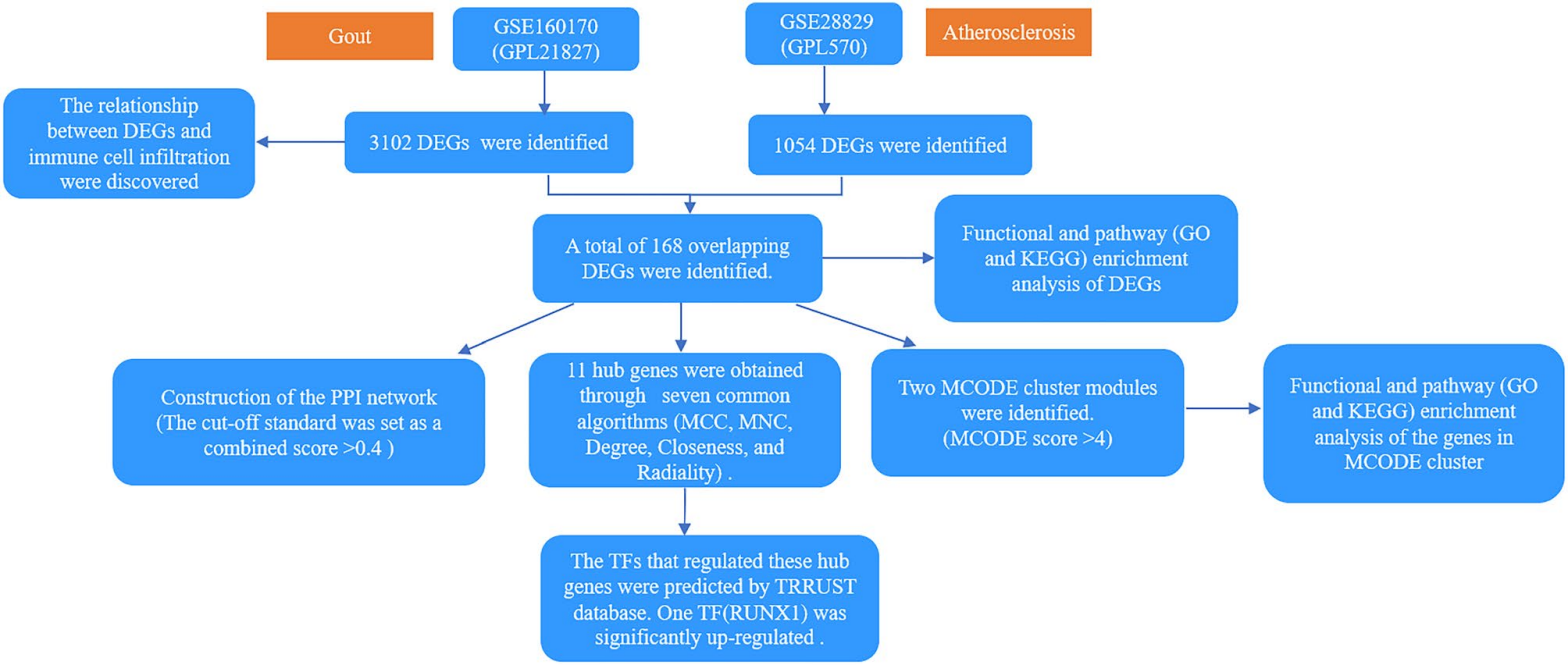
The flowchart of the overall study.

“Gout’’ or “atherosclerosis” were used as key words for the expression profiling of gout or atherosclerosis in the GEO database, which is a public repository database^9^. Datasets, including peripheral blood mononuclear cells from gout or plaque biopsies from atherosclerosis, were used. Finally, two datasets, namely, GSE160170 (GPL21827) and GSE28829 (GPL570) were selected. GSE160170 includes peripheral blood mononuclear cells from six gouty patients and six healthy controls^10^. GSE28829 (GPL570) included plaque biopsies from 13 intimal thickening and 16 thick fibrous cap atheroma lesions^11^. In the original articles generating these two datasets (GSE160170 and GSE28829), written informed consents were obtained from all the enrolled participants. The overall flowchart of this research is shown in Fig. 1.

### Identification of DEGs

The row expression data of GSE160170 and GSE28829 were analyzed. DEGs between the disease and healthy control groups were obtained using the online web-based tool GEO2R, an R-based web application that helps users analyze GEO data^12^. Adjusted P value < 0.01 was considered statistically significant. Overlapping DEGs of gout and atherosclerosis were detected with the online tool Draw Venn Diagram (http://bioinformatics.psb.ugent.be/webtools/Venn/).

### Functional and pathway enrichment analyses

Gene ontology (GO) enrichment and Kyoto Encyclopedia of Genes and Genomes (KEGG) analyses were performed for the identified overlapping DEGs. R packages (clusterProfile, ggplot2 and GOplot) were used for the analyses^13^.

### Construction of a PPI network

The online tool STRING (https://string-db.org) was used for the construction of a PPI network using the common DEGs. The cut-of standard was set as a combined score > 0.4^14^. In general, the interaction scores in STRING are meant to express an approximate confidence, on a scale of zero to one, of the association being true, given all the available evidence^14–16^. Then, the results were visualized with CytoScape software. Molecular complex detection (MCODE) V1.5.1, which is a plug-in of CytoScape, was used in identifying significant modules (MCODE score ≥ 4)^17^. Moreover, the hub genes were selected using CytoHubba, which is another plug-in of CytoScape, according to the number of associations with other genes in the PPI network^18^. Hub genes were selected by five common algorithms (MCC, MNC, Degree, Closeness, and Radiality).

### Prediction of TFs

A database for the prediction of transcriptional regulatory networks, transcriptional Regulatory Relationships Unraveled by Sentence Based Text Mining (TRRUST), was used in predicting TFs that regulate hub genes^19^. Adjusted P value of < 0.05 was considered significant^20^.

### Immune infiltration of gout related DEGs

Single-sample GSEA (ssGSEA) was utilized for immune infiltration analysis of gout related DEGs in GSE160170^21^. Marker genes of immune cell types for ssGSEA were obtained from Charoentong P et al.^22^. Infiltration levels for different immune cell types were quantified using the ssGSEA implementation by the R package “gsva”^23^. Moreover, the R package “estimate” was used to infer the fraction of immune cells (ImmuneScore) in gout samples based on given gene expression profile in FPKM or normalized log2 transformed values^24^. ssGSEA scores for each individual immune cell type were used to calculate immune infiltration score^25^.

## Results

### Identification of common DEGs

**Figure 2:**
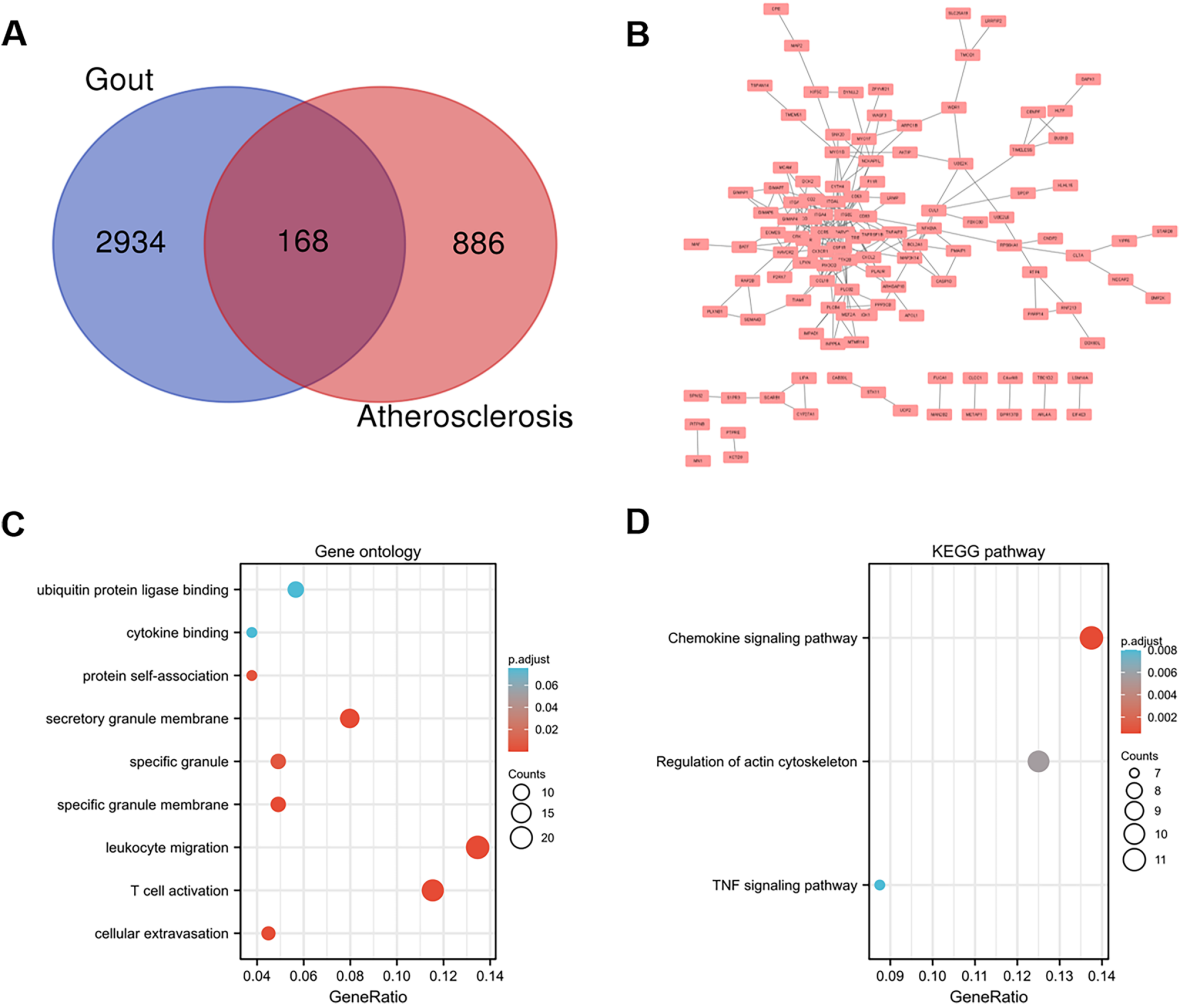
Venn diagram, protein–protein interaction network, and functional enrichment of DEGs. (**A**) Venn diagram of common DEGs from the two datasets. (**B**) The interaction network between proteins coded by DEGs. The enrichment analysis results of GO (**C**) and KEGG (**D**) pathway. Adjusted P value < 0.05 was considered significant.

DEGs were identified after the microarray results were standardized. A total of 3102 DEGs were found in the gout dataset (GSE160170), and 1054 DEGs were found in the atherosclerosis dataset (GSE28829). A total of 168 common DEGs were found after the integration of the DEGs (Fig. 2A).

### PPI network construction and functional analyses

The PPI network for the 168 DEGs was constructed after the common DEGs were imported to STRING (Fig. 2B). GO and KEGG analyses were used in analyzing the 168 common DEGs (Fig. 2C, D and Table 1)^26–28^. The biological process acted primarily on leukocyte migration, T cell activation, and cellular extravasation based on GO enrichment. These proteins were primarily located in specific granule membrane, secretory granule membrane, and specific granule. With regard to molecular functions, the proteins played a role in protein self-association (Fig. 2C and Table 1). According to KEGG pathway analysis, these proteins were primarily involved in chemokine signaling pathway, regulation of actin cytoskeleton, and TNF signaling pathway (Fig. 2D).

**Table 1:** GO and KEGG analysis of DEGs.

	ID	Description	GeneRatio	BgRatio	p. adjust
BP	GO:0050900	Leukocyte migration	21/156	499/18670	3.18e–06
BP	GO:0042110	T cell activation	18/156	464/18670	9.18e–05
BP	GO:0045123	Cellular extravasation	7/156	61/18670	6.71e–04
BP	GO:0072676	Lymphocyte migration	8/156	111/18670	0.002
BP	GO:0002446	Neutrophil mediated immunity	16/156	499/18670	0.002
CC	GO:0035579	Specific granule membrane	8/163	91/19717	1.80e–04
CC	GO:0030667	Secretory granule membrane	13/163	298/19717	1.80e–04
MF	GO:0043621	Protein self-association	6/159	56/17697	0.004
KEGG	hsa04062	Chemokine signaling pathway	11/80	192/8076	5.50e–04
KEGG	hsa04810	Regulation of actin cytoskeleton	10/80	218/8076	0.006
KEGG	hsa04668	TNF signaling pathway	7/80	112/8076	0.008
KEGG	hsa04070	Phosphatidylinositol signaling system	6/80	97/8076	0.016
KEGG	hsa05135	Yersinia infection	7/80	137/8076	0.016

*BP* biological process group, *CC* cellular component group, *MF* molecular function group, *KEGG* Kyoto Encyclopedia of Genes and Genomes.

### MCODE cluster modules identification and functional analyses of cluster genes

**Figure 3:**
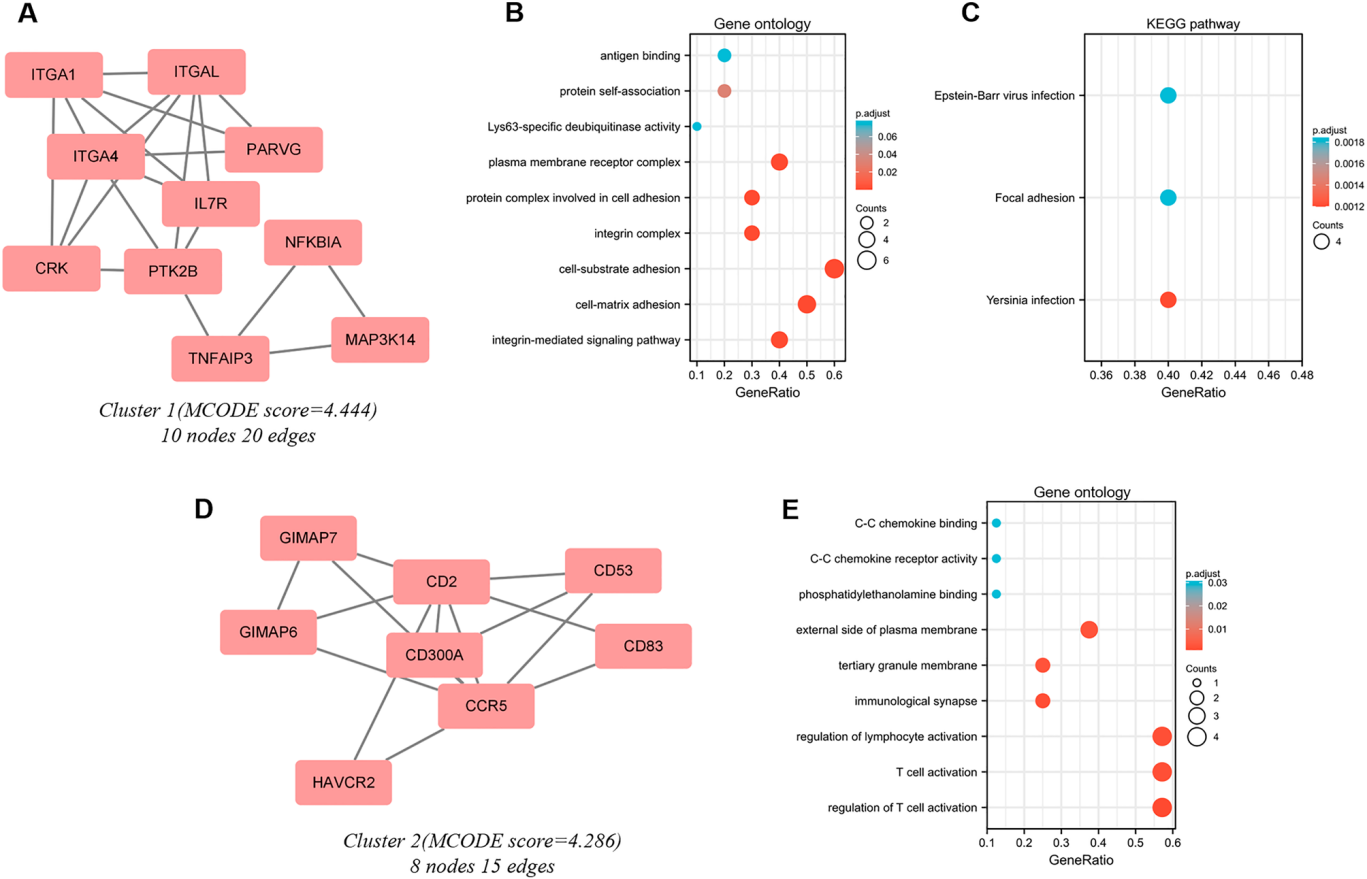
Cluster modules extracted by MCODE and enrichment analyses of the modular genes. Two significant gene clustering modules extracted by MCODE. Cluster 1 (**A**) had higher cluster score (MCODE score = 4.444), followed by cluster 2 (**D**) (MCODE score = 4.286). GO (**B**) and KEGG (**C**) enrichment analyses of the modular genes in cluster 1. GO (**E**) enrichment analysis of the modular genes in cluster 2. Adjusted P value < 0.05 was considered significant.

Significant modules of the PPI network were identified by MCODE with a threshold of 4. Two modules with MCODE scores of ≥ 4 are illustrated in Figs. 3A and D. One cluster (MCODE score = 4.444) had 10 nodes and 20 edges (Fig. 3A). GO analysis showed that the proteins in the cluster were related to protein self-association, antigen binding, and Lys63-specific deubiquitinase activity. (Fig. 3B). KEGG pathway analysis showed that these proteins were primarily involved in Epstein − Barr virus infection, focal adhesion, and Yersinia infection (Fig. 3C). The other cluster (MCODE score = 4.286) had 8 nodes and 15 edges (Fig. 3D). GO analysis showed that the proteins in the cluster were related to regulation of lymphocyte activation, T cell activation, and regulation of T cell activation (Fig. 3E) and there seemed no significant KEGG pathway been clustered in this module.

### Hub gene selection and analysis

**Figure 4:**
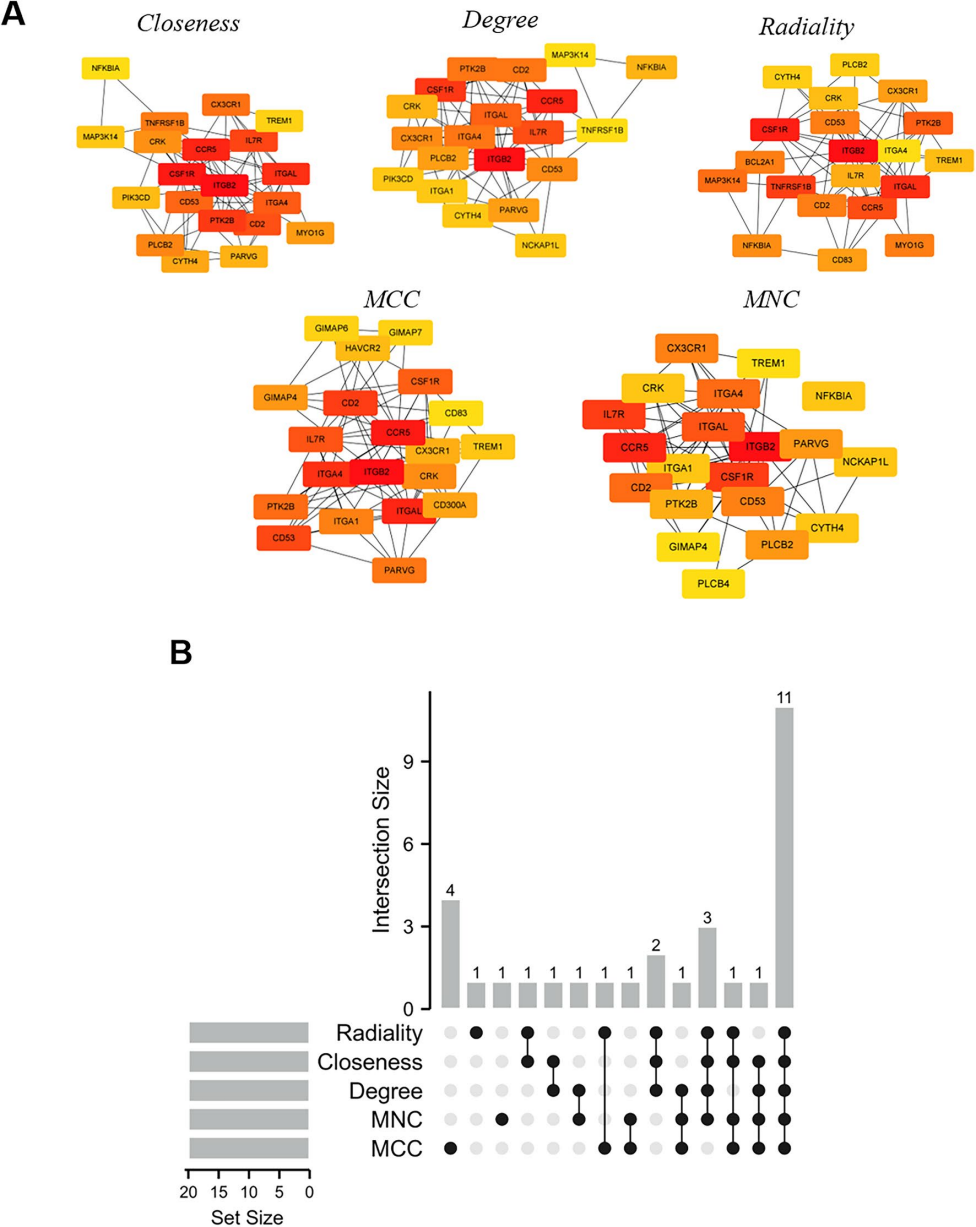
Hub genes identified by different algorithms and UpSet diagram. (**A**) Hub gene identified by five different algorithms. (**B**) The UpSet diagram showed that the five algorithms screened 11 overlapping hub genes.

**Figure 5:**
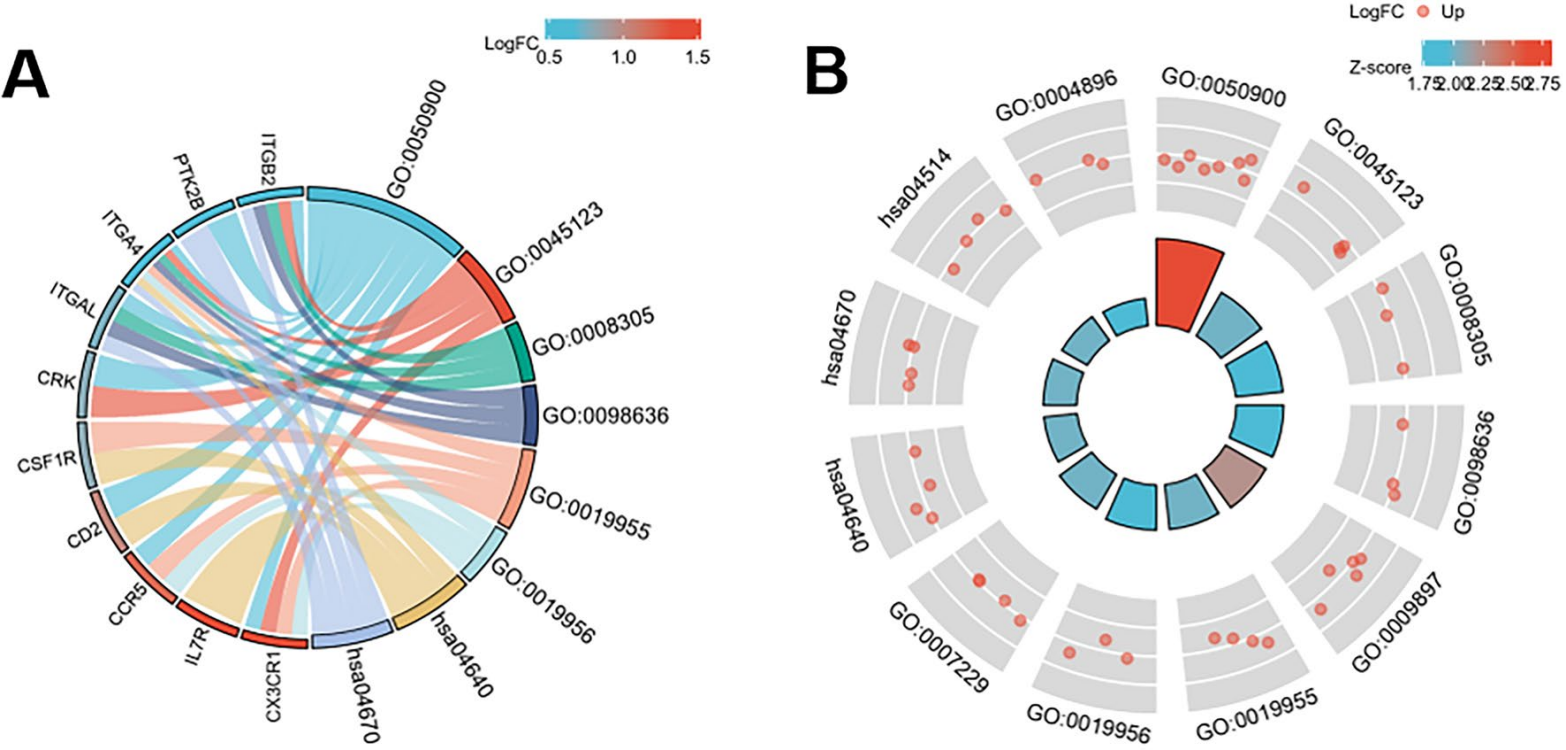
GO and KEGG enrichment analysis of the hub genes. GO:0050900, leukocyte migration; GO:0045123, cellular extravasation; GO:0008305, integrin complex; GO:0098636, protein complex involved in cell adhesion; GO:0019955, cytokine binding; GO:0019956, chemokine binding; hsa04640, Hematopoietic cell lineage; hsa04670, Leukocyte transendothelial migration.

The top 20 hub genes were calculated using the five algorithms of the plug-in CytoHubba (Fig. 4A). After the intersection of the UpSet diagram was determined, 11 common hub genes were discovered, namely, integrin subunit beta 2(ITGB2), C–C motif chemokine receptor 5 (CCR5), integrin subunit alpha L(ITGAL), integrin subunit alpha 4 (ITGA4), CD2 molecule (CD2), CD53 molecule (CD53), interleukin 7 receptor (IL7R), colony stimulating factor 1 receptor (CSF1R), protein tyrosine kinase 2 beta(PTK2B), CRK proto-oncogene, adaptor protein(CRK), and C-X3-C motif chemokine receptor 1 (CX3CR1,Fig. 4B). Table 2 shows their full names and related functions. GO analysis showed that the genes were mainly involved in cytokine binding, chemokine binding, and cytokine receptor activity. KEGG pathway analysis revealed that the hub genes were primarily involved in hematopoietic cell lineage, leukocyte transendothelial migration, and cell adhesion molecules (Fig. 5A and B). The expression of identified hub genes in the datasets of gout and atherosclerosis is shown in Table 3. Except CRK, the other hub genes were all significantly upregulated in both gouty and atherosclerotic patients.

**Table 2:** The details of the hub genes.

Gene symbol	Full name	Accession number	Function
ITGB2	Integrin subunit beta 2	HGNC:6155	This gene encodes an integrin beta chain. Integrins are integral cell-surface proteins that participate in cell adhesion as well as cell-surface mediated signaling^44^
CCR5	C–C motif chemokine receptor 5	HGNC:1606	This protein is expressed by T cells and macrophages, and is known to be an important co-receptor for macrophage-tropic virus^45^
ITGAL	Integrin subunit alpha L	HGNC:6148	This integrins I-domain containing alpha integrin combines with ITGB2 to form the integrin lymphocyte function-associated antigen-1 (LFA-1), which is expressed on all leukocytes^46^
ITGA4	Integrin subunit alpha 4	HGNC:6140	ITGA4 associates with a beta 1 or beta 7 subunit to form an integrin that may play a role in cell motility and migration^47^
CD2	CD2 molecule	HGNC:1639	CD2 interacts with LFA3 (CD58) on antigen presenting cells to optimize immune recognition^48^
CD53	CD53 molecule	HGNC:1686	CD53 is a cell surface glycoprotein that is known to complex with integrins. It contributes to the transduction of CD2-generated signals in T cells and natural killer cells and has been suggested to play a role in growth regulation^49^
IL7R	Interleukin 7 receptor	HGNC:6024	The function of IL7R requires the interleukin 2 receptor, gamma chain (IL2RG), which is a common gamma chain shared by the receptors of various cytokines, including interleukins 2, 4, 7, 9, and 15^50^
CSF1R	Colony stimulating factor 1 receptor	HGNC:2433	CSF1R controls the production, differentiation, and function of macrophages and mediates most if not all of the biological effects of CSF1^51^
PTK2B	Protein tyrosine kinase 2 beta	HGNC:9612	PTK2B is involved in calcium-induced regulation of ion channels and activation of the map kinase signaling pathway^52^
CRK	CRK proto-oncogene, adaptor protein	HGNC:2362	CRK binds to several tyrosine-phosphorylated proteins^53^
CX3CR1	C-X3-C motif chemokine receptor 1	HGNC:2558	CX3CR1 is a receptor for fractalkine and is a coreceptor for HIV-1, and some variations^54^

**Table 3:** The expression of identified hub genes in gout and atherosclerosis datasets.

Hub gene	Gout		Atherosclerosis	
	LogFC	*P. adj*	LogFC	*P. adj*
ITGB2	0.472585	0.00227	2.118971	0.0000543
CCR5	1.203069	0.000409	0.477587	0.0000996
ITGAL	0.750841	0.0088	0.4475	0.0002744
ITGA4	0.558308	0.000694	0.964228	0.0020748
CD2	1.023166	9.79E-06	0.449125	0.0028765
CD53	0.852588	0.000743	1.66447	0.000162
IL7R	1.484619	1.26E-06	1.026407	0.0063337
CSF1R	0.79756	0.00274	1.172456	0.0009781
PTK2B	0.523546	0.00863	0.481643	0.0056084
CRK	0.786725	4.06E-05	-0.29272	0.0023219
CX3CR1	1.524042	0.000341	1.132646	0.0055004

### Prediction and verification of TF

**Figure 6:**
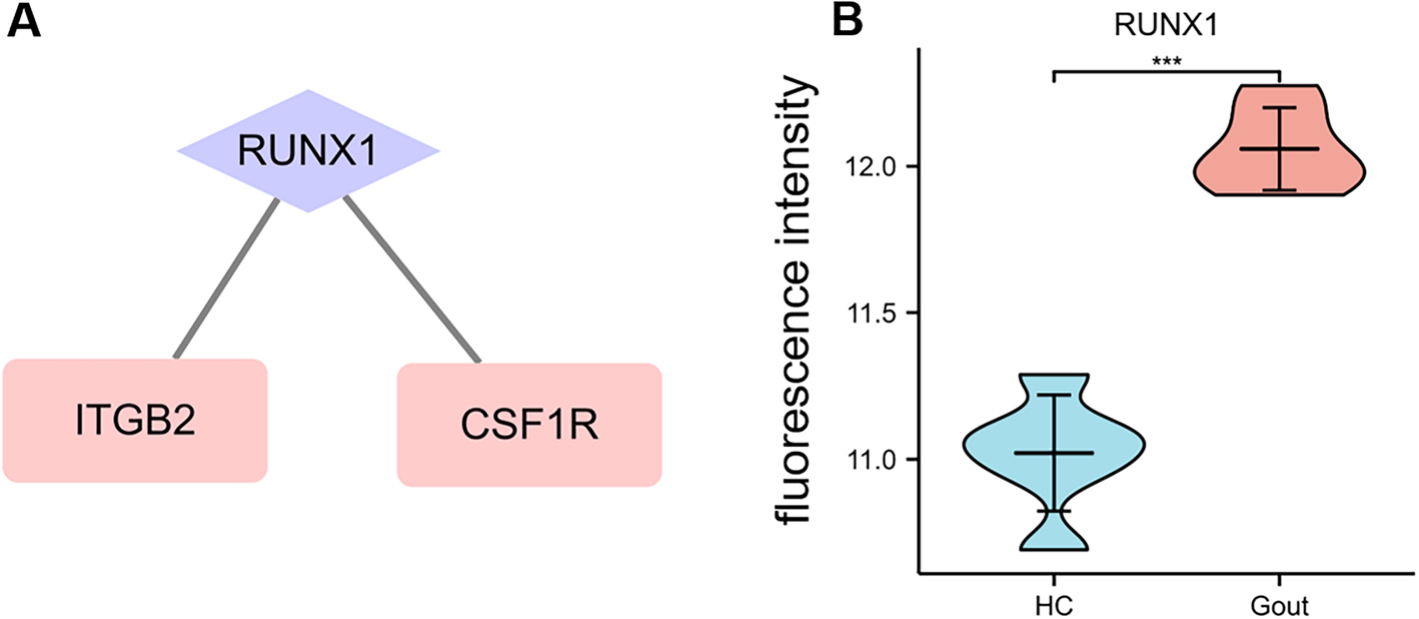
TF regulatory network and its expression in GSE160170. (**A**) TF regulatory network. (**B**) The expression level of RUNX1 in GSE160170. ***P < 0.001.

One TF that may regulate the expression of the hub genes were identified on the basis of the TRRUST database (Fig. 6A and Table 4). RUNX family transcription factor 1 (RUNX1) were predicted to have the capability to regulate two hub genes (including ITGB2 and CSF1R) by acting as a TF. The expression of RUNX1 were found to be significantly increased in gouty patients (Fig. 6B).

**Table 4:** Key transcription factors (TFs) of hub genes.

Key TFs	Description	P-value	Genes
RUNX1	Runt-related transcription factor 1	0.000238	CSF1R, ITGB2

### Immune cell infiltration analyzed by ssGSEA

**Figure 7:**
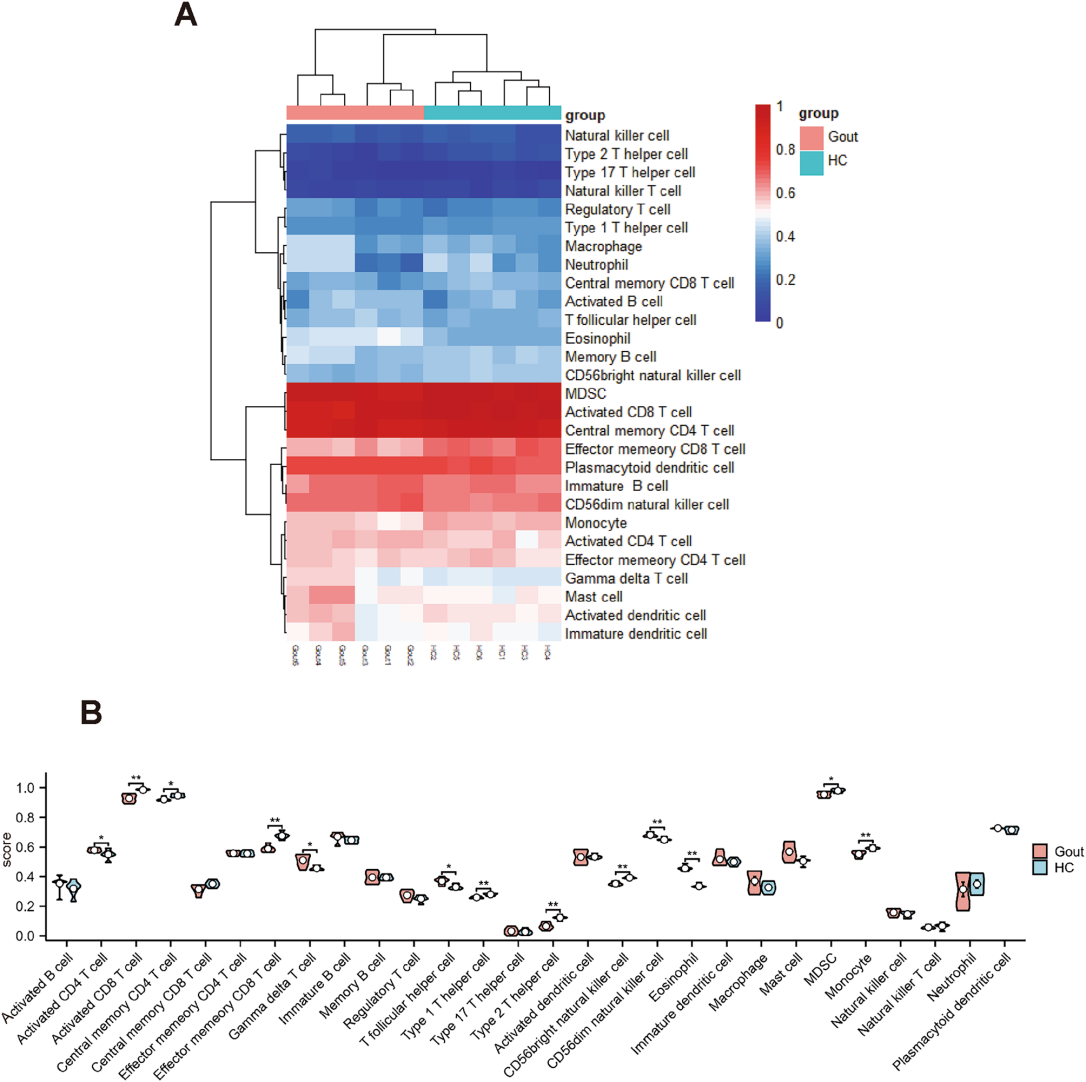
Evaluation and visualization of immune cell infiltration. (**A**) Heat map of 28 types of immune cell. (**B**) Violin diagram of the proportion of 28 types of immune cells.

ssGSEA was employed to measure the per sample infiltration levels of 28 immune cell types^22^. Correlation analysis revealed that gouty patients had higher level of activated CD4 T cells, gamma delta T cells, T follicular helper cell, CD56dim natural killer cells, and eosinophil (P < 0.05, Wilcoxon rank-sum test, Fig. 7A and B).

## Discussion

In this study we tried to identify the underlying mechanisms of gout complicated with atherosclerosis. 168 DEGs and 11 hub genes were detected. Among the 11 hub genes, ten genes except CRK were all upregulated in both diseases. Functional enrichment analyses revealed that the genes were significantly enriched in chemokine signaling pathway, regulation of actin cytoskeleton, and TNF signaling pathway. In addition, one TF, RUNX1, was predicted to play a role in the pathogenesis process. Meanwhile, Immune cell infiltration result indicated that gouty patients had higher level of activated CD4 T cells, gamma delta T cells, T follicular helper cell, CD56dim natural killer cells, and eosinophil compared to healthy controls.

Gout and atherosclerosis are two different types of inflammatory diseases. Through analyzing the common DEGs, the common pathogenic pathways can be identified and inhibit the common pathogenesis pathway may have a multiplier effect in treating primary gout patients complicated with atherosclerosis. According to our results, chemokine signaling pathway, regulation of actin cytoskeleton, and TNF signaling pathway seemed to be activated in gout complicated with atherosclerosis. Chemokines are mainly responsible for the recruitment and movement of cells and involved in the proceeding of atherosclerosis^29^. Meanwhile, chemokines play vital roles in the process of gout flare and remission^30^.Therefore, the activation chemokines signaling pathway may contribute simultaneously to atherosclerosis and gout. Regulation of actin cytoskeleton is also found to be activated. In 2016, Rong Liu et al. found that altering cytoskeleton-based functions by depleting calponin 2 could attenuate the development of atherosclerosis^31^. Furthermore, it is discovered that when cells are well expanded, dephosphorylation of YAP would be triggered through the cellular cytoskeleton reorganizes and develops increased number of stress fibers^31^. Thus, changes in cell shape leading to reorganization of actin cytoskeleton can regulate growth and proliferation, making cytoskeleton regulation a potential intervention for atherosclerosis^31^. TNF, one of the proinflammatory cytokines, play an important role in the pathophysiology of the inflammatory arthritis including gout and are associated with the induction and maintenance of the atherosclerosis^32,33^.Two studies reported that TNF antagonists may have a beneficial effect on preventing the progression of subclinical atherosclerosis^33,34^. Wei Gao et al. discovered the underlying mechanism and found that exosomes derived from mature dendritic cells increase atherosclerosis via membrane TNF mediated NF-kB pathway^35^. Therefore, TNF signaling pathway may be a promising treatment target for patients with gout complicated with atherosclerosis.

Moreover, our study identified hub genes by using five common algorithms. Among those of the 11 identified hub genes, two hub genes, including ITGB2 and CSF1R, were predicted to be regulated by a TF, RUNX1. In addition, the expression of RUNX1 is found to be significantly upregulated in gouty patients. ITGB2 belongs to integrin beta chain. Integrins are integral cell-surface proteins that participate in cell adhesion as well as cell-surface mediated signaling. It is reported that under the stimulation of inflammation and thrombus, ITGB2 can involve in the adhesion of neutrophils and monocytes to endothelial cells^36,37^. Meanwhile, ITGB2 was found to be robustly upregulated in the arterial plaques and all plaque location. Therefore, ITGB2 may serve as a common target for gout with atherosclerosis. Meanwhile, CSF1R is also found to be significantly upregulated as a hub gene in our study. CSF1R controls the production, differentiation, and function of macrophages and mediates most of the biological effects of CSF1. Previous study showed that miR-155 are attributable to the inhibition of macrophage proliferation by suppressing CSF1R in early atherosclerosis^38^. In addition, CSF1R was demonstrated to be negatively associated with the level of HDL-C, indicating the link between CSF1R and atherosclerosis^39^. ITGB2 and CSF1R were found to be regulated by the same TF, RUNX1. RUNX1 is demonstrated to be elevated in atherosclerotic aortas and gouty patients^40^. RUNX1 has long been considered as a potential therapeutic target in atherosclerosis. Our study discovered that RUNX1 could regulate two hub genes. Hence, downregulating RUNX1 would possibly alleviate the atherogenesis in gouty patients.

Immune infiltration of patients with gout was also investigated. Correlation analysis revealed that gouty patients had higher level of activated CD4 T cells, gamma delta T cells, T follicular helper cell, CD56dim natural killer cells, and eosinophil. The importance of CD4 + T cells in atherogenesis has been highlighted by animal studies showing that transfer of CD4 + T cells aggravates, whilst CD4 + T cell deficiency attenuates atherosclerosis^41^. In the meantime, previous study found that the increased frequencies of T follicular helper cell may suggest the inflammatory response and atherosclerosis progression^42^. CD56dim natural killer cells was a subset of natural killer cells and is best-known by their cytotoxic functions. However, its role in atherosclerosis is still uncovered. Concerning eosinophil, one study demonstrated that eosinophils promote arterial thrombosis by eosinophil extracellular traps formation and major basic protein release resulting in platelet activation^43^. Therefore, eosinophils are a promising new target in the prevention and therapy of atherosclerosis and thrombosis. However, there were several proatherogenic immune cells found to be decreased in gouty patients, including CD8 T cells, type 1 T helper cells, and type 2 T helper cells. This result was in accordance with a previous study reporting that patients with gout have short telomeres, while the differences of immune cells in their study were not significant^8^. The exact reason for this phenomenon was not fully uncovered. This may result from the specific properties of uric acid, which need to be further studied.

In the interpretation of our results, the following limitation require careful discussion. It was not known whether enrolled gout patients were in acute gout attack. The heatmap clustering shows two types of gout patients, one of which is more like the control group, hence, these patients may be in interval period. As a result, a more precise designed and larger-population study is needed to further assess immune infiltration result.

## Conclusion

To sum up, our study tried to identify the possible hub genes and TFs, which may be promising treatment targets for patients with gout complicated with atherosclerosis. In the meantime, we discovered the immune infiltration of gout. 11 genes were identified as hub genes. KEGG pathway enrichment analyses revealed that the common DEGs were significantly enriched in chemokine signaling pathway, regulation of actin cytoskeleton, and TNF signaling pathway. In addition, one TF, RUNX1, was predicted to play a role in the pathogenesis process. Immune cell infiltration result indicated that gouty patients had higher level of activated CD4 T cells, gamma delta T cells, T follicular helper cell, CD56dim natural killer cells, and eosinophil. These immune cells may play a key role in the development of gout, and further exploration of these immune cells may determine the targets of immunotherapy in gouty patients.

## Data Availability

The datasets generated and/or analyzed during the current study are available in the GEO repository. It is a public free repository database, which stores a large number of gene functions and expressions. The working links are as following, GSE160170 (https://www.ncbi.nlm.nih.gov/geo/query/acc.cgi?acc=GSE160170) and GSE28829 (https://www.ncbi.nlm.nih.gov/geo/query/acc.cgi?acc=GSE28829).
